# Mechanical ventilation with high tidal volume induces inflammation in patients without lung disease

**DOI:** 10.1186/cc8919

**Published:** 2010-03-18

**Authors:** Roselaine Pinheiro de Oliveira, Marcio Pereira Hetzel, Mauro dos Anjos Silva, Daniele Dallegrave, Gilberto Friedman

**Affiliations:** 1 Central Intensive Care Unit, Complexo Hospitalar Santa Casa, Rua Prof. Annes Dias, 295, Porto Alegre, 90020-090, Brazil; 2 Hospital de Clínicas de Porto Alegre, Universidade Federal do Rio Grande do Sul, Ramiro Barcelos n° 2.350, Porto Alegre, 90035-903, Brazil

## Abstract

**Introduction:**

Mechanical ventilation (MV) with high tidal volumes may induce or aggravate lung injury in critical ill patients. We compared the effects of a protective versus a conventional ventilatory strategy, on systemic and lung production of tumor necrosis factor-α (TNF-α) and interleukin-8 (IL-8) in patients without lung disease.

**Methods:**

Patients without lung disease and submitted to mechanical ventilation admitted to one trauma and one general adult intensive care unit of two different university hospitals were enrolled in a prospective randomized-control study. Patients were randomized to receive MV either with tidal volume (V_T_) of 10 to 12 ml/kg predicted body weight (high V_T _group) (n = 10) or with V_T _of 5 to 7 ml/kg predicted body weight (low V_T _group) (n = 10) with an oxygen inspiratory fraction (FIO_2_) enough to keep arterial oxygen saturation >90% with positive end-expiratory pressure (PEEP) of 5 cmH_2_O during 12 hours after admission to the study. TNF-α and IL-8 concentrations were measured in the serum and in the bronchoalveolar lavage fluid (BALF) at admission and after 12 hours of study observation time.

**Results:**

Twenty patients were enrolled and analyzed. At admission or after 12 hours there were no differences in serum TNF-α and IL-8 between the two groups. While initial analysis did not reveal significant differences, standardization against urea of logarithmic transformed data revealed that TNF-α and IL-8 levels in bronchoalveolar lavage (BAL) fluid were stable in the low V_T _group but increased in the high V_T _group (*P *= 0.04 and *P *= 0.03). After 12 hours, BALF TNF-α (*P *= 0.03) and BALF IL-8 concentrations (*P *= 0.03) were higher in the high V_T _group than in the low V_T _group.

**Conclusions:**

The use of lower tidal volumes may limit pulmonary inflammation in mechanically ventilated patients even without lung injury.

**Trial Registration:**

Clinical Trial registration: NCT00935896

## Introduction

Clinical studies suggest that mechanical ventilation (MV) can modify inflammatory responses in patients with acute lung injury. In such patients, with existing pulmonary and systemic inflammation, ventilation with tidal volumes (V_T_) of 10 to 15 mL/kg predicted body weight and low-to-moderate levels of positive end expiratory pressure (PEEP) was associated with increased intraalveolar and systemic levels of inflammatory mediators [1]. In contrast, mechanical ventilation with moderate-to-high levels of PEEP and low V_T _of approximately 6 mL/kg predicted body weight assured adequate gas exchange, decreased intraalveolar and systemic mediator levels, and improved outcome [1-4]. Experimental data suggest that mechanical ventilation with higher V_T _and zero end-expiratory pressure (ZEEP) induces not only cytokine release but also translocation of cytokines from the lungs to the systemic circulation and even vice versa [5-7]. The clinical repercussion of these studies is uncertain because unphysiologically large V_T_s and no PEEP were generally compared to low V_T _and PEEP.

In contrast to patients with acute lung injury having a continuing systemic inflammatory reaction, it is not clear if MV by itself can initiate lung inflammation. Observational studies have showed that a lung inflammatory response could be induced after conventional and prolonged mechanical ventilation in a mixed population of critically ill patients [8,9]. Retrospective observations suggest that higher V_T_s may be deleterious after prolonged ventilation or major surgery [9,10]. Three randomized studies on surgical patients suggested that a pulmonary inflammatory response could be induced by a short-term mechanical ventilation (up to 10 hours) even in lungs without pre-existing injury [11-13]. However, most studies used high V_T _and no PEEP in comparison to low V_T _and PEEP. Thus, it is not known whether short-term mechanical ventilation with PEEP and moderate to high V_T _could induce signs of pulmonary or/and a systemic inflammation.

We hypothesized that lung-protective mechanical ventilation with lower tidal volumes, as compared to conventional mechanical ventilation induces less inflammation in critically ill patients without evidence of lung disease. To test this hypothesis, we measured tumour necrosis factor-alpha and interleukin-8 in the plasma and in the bronchoalveolar lavage (BAL) while patients were mechanically ventilated with lung-protective or conventional strategies.

Grant CAPES-PROF, Faculdade de Medicina - Federal University of Rio Grande do Sul.

## Materials and methods

### Patient selection

Twenty patients admitted to a clinical-surgical (Complexo Hospitalar Santa Casa) and trauma (Hospital de Pronto Socorro) ICU were enrolled in a randomized and prospective study. Approval of both institutional Ethics Committees for the study protocol was obtained and all patients (or next of kin) gave written informed consent before inclusion in the study.

Inclusion criteria were: 1) age ≥16 years; 2) anticipated survival >24 hours; 3) need for mechanical ventilation for at least 12 hours and 4) hemodynamic stability (MAP >65 mmHg, HR <100 beats/minute, diuresis >1 ml/kg/h, no catecholamine requirement or fluid challenge).

Exclusion criteria included thoracic surgical procedures, use of immunosuppressive medication, recent infections, previous thromboembolic disease, recent ventilatory support, and participation in another clinical trial. Absence of lung disease was defined by the following clinical criteria: (a) no evidence of respiratory infection (white blood cell count <10 × 10^3^/μl, temperature >38°C, purulent sputum), (b) normal chest roentgenogram, (c) ratio between arterial oxygen tension and inpired oxygen tension (PaO_2_/FIO_2_) >300, (d) and a normal clinical respiratory history.

Patients were on mechanical ventilation for a maximum of 12 hours at the time of initiating one of the two randomized MV strategies, including the surgical period. On ICU admission, the following standard ventilation protocol was applied: patients were continuously sedated (benzodiazepines and/or opioids), remained supine and were ventilated with intermittent positive pressure ventilation, assist/control mode on a Siemens Elema 900C Servo ventilator (Solna, Sweden). V_T_, respiratory rate, and fraction of inspired oxygen (FIO_2_) were adjusted to maintain arterial oxygen saturation >90%, PaCO_2_ of 35 to 45 mmHg and pH >7.25. PEEP was kept at 5 cmH_2_O. The inspiratory:expiratory (I:E) ratio was 1:2. All ventilator circuits were equipped with a heat-moisture exchanger.

Disease severity was scored with the Acute Physiology and Chronic Health Evaluation (APACHE) II scoring system [14].

### Measurements and study protocol

Immediately after ICU admission, once all inclusion and exclusion criteria were met and consent obtained, 20 patients were randomly (opaque sealed envelopes) assigned to receive mechanical ventilation in volume-controlled mode either with V_T _of 10 to 12 ml/kg predicted body weight (high V_T _group, n = 10) or with V_T _of 5 to 7 ml/kg predicted body weight (low V_T _group, n = 10) with an inspiratory fraction of oxygen (FIO_2_) set at the minimal level at which an arterial oxygen saturation of >90% and minimal PEEP (4 to 5 cmH_2_O). I:E ratio was 1:2. The predicted body weight of male patients was calculated as equal to 50 + 0.91(centimeters of height-152.4); that of female patients was calculated as equal to 45.5 + 0.91(centimeters of height-152.4) [4]. Baseline serum and BAL samples for tumor necrosis factor-alpha (TNF-α) and interleukin-8 (IL-8) measurements were taken. Additional serum and BAL samples were obtained 12 h after randomization for comparison. All blood and BAL samples were collected and handled by the same investigator. All patients remained supine throughout the study period. The following ventilatory variables were measured at baseline and 12 hours: tidal volume (V_T_), minute ventilation (VE), inspiratory time (TI), expiratory time (TE), positive end-expiratory pressure (PEEP), peak inspiratory pressure (Ppeak), and plateau pressure after end-inspiratory pause (Pplateau) [3].

All patients received sedation and analgesia to keep them comfortable while on mechanical ventilation. Patients were not left on ventilation for the study and one patient was extubated and excluded from the analysis before protocol initiation.

### Bronchoalveolar lavage (BAL)

BAL was performed by instillating 100 ml sterile isotonic saline (five aliquots of 20 ml) in segments of the right lower lobe and sequentially suctioned; 30% to 50% of this aliquot was recovered. The first aliquot was discharged. During bronchoscopy FIO_2 _was kept at 100%. Lavage fluids were filtered through sterile gauze filters, collected on ice, and immediately centrifuged at 1,500 *g *for 10 minutes. Supernatant aliquots were kept frozen at -40°C for subsequent analysis.

### Blood measurements

Venous ethylenediaminetetraacetic acid (EDTA) blood samples from fresh puncture sites of 10 ml were obtained and immediately centrifuged at 1,500 *g *for 10 minutes; the plasma was aspirated and stored at -40°C.

### Cytokines measurements

Commercially available ELISA assays were used to measure BAL and plasma levels of human interleukin 8 (IL-8), tumor necrosis factor alpha (TNF-α) (R&D Systems, Minneapolis, MN, USA). All enzyme-linked immunosorbent assays were performed according to the manufacturers' guidelines. All samples from one patient were analyzed in the same assay run. The samples were measured in duplicates by the same technician who was blinded to ventilation strategy. Samples were assayed for each 10 patients and a randomization code broken. The sensitivities of the test kits were as follows: IL-8: 1.5 pg/mL and TNF-α: 0.5 pg/mL.

### Standardization of cytokine concentrations in BAL with urea

The technique of BAL is based on the concept that aliquots of sterile normal saline solution infused through the bronchoscope mix with epithelial lining fluid (ELF). The use of urea to quantify the amount of ELF recovered by BAL is based on the knowledge that urea is freely diffusible through most body compartments, including the lungs. Urea concentration was measured in BAL fluid and blood and ELF volume calculated according to the formula described below [15]. In this context, if the concentration of urea in plasma is known and the quantity of urea in a lavage sample is measured, the volume of recovery ELF can be calculated as:

Volume of ELF (mL) = total amount urea in BALF (mg)/concentration of urea in plasma (mg/mL) (I)

The cytokine concentrations in the ELF were then calculated as:

Cytokine of ELF (pg/mL) = total amount of cytokine in BALF (pg/mL)/volume of ELF (II)

### Statistical analysis

The required sample size was calculated from previous studies on ventilatory strategies in patients during major surgeries [11,16] and after the first 10 patients' analyses. To detect differences in the time course of plasma TNF-α between the ventilatory settings with respect to the two groups with the given two-tailed parallel design at a significance level of 5% (a = 0.05) with a probability of 80% (b = 0.20) based on an estimated difference of 0.76 of the parameter's mean standard deviation, the number of patients to be studied in each group was 10. Data were tested for normal distribution with the Kolmogorov-Smirnov test. Differences within groups were analyzed with a *t*-test or Wilcoxon signed-rank test for paired samples. Student *t*-test or Mann-Whitney U test were used to compare the changes over time between the two randomization groups as appropriate. Logarithmic transformation of BAL urea cytokines values was also used to stabilize the variance and to permit the application of a parametric test. Differences were considered to be statistically significant at the level of *P *< 0.05. Results are expressed as mean ± standard deviation or median (25^th ^to 75^th ^percentiles).

## Results

The two groups of patients did not differ significantly in demographic or clinical data (Table 1). For the nine surgical patients, the median surgery duration was 470 minutes (435 minutes to 480 minutes). Three patients of each group received up to two packed red blood cells during surgery. Blood transfusions or surgical interventions were not required during the 12 hours study period.

**Table 1:** Demographic and clinical data

	High V_T _ (n = 10)	Low V_T _(n = 10)	*P *value
Age (years)	52 ± 18	46 ± 18	0.43
Gender: M/F	7/3	9/1	0.58
APACHE II	16.0 ± 8.6	13.2 ± 6.5	0.42
Scheduled surgery			
• Gastrointestinal	3	4	
• Vascular	1		
• Other	1		
			
Clinical diagnosis			
• Head trauma	2	4	
• Stroke	2	2	
• Other	1		
			
Lenght of ICU stay (days)	6.6 ± 5.0	7.7 ± 7.6	0.71
Duration of MV (days)	3 [1 to 5]	7 [1 to 9]	0.65
28^th ^Mortality rate (%)	40	30	1.00

V_T_, tidal volume; APACHE, Acute Physiology and Chronic Health Evaluation; MV, mechanical ventilation.

Ventilatory and blood gases parameters are shown in Table 2. As expected, V_T_, plateau pressure and peak pressure became higher in the high V_T _group throughout the 12 h observation time. Although not significantly, PaCO_2 _and HCO_3 _values were lower in the high V_T _group enough to keep pH values stable after 12 h.

**Table 2:** Ventilatory parameters and arterial blood gases

	*High V*_*T *_ *Group*		*Low V*_*T *_ *Group*	
***Time***	**Zero**	**12 h**	**Zero**	**12 h**
V_T _(PBW) (ml)	11.3 ± 0.8**	11.9 ± 0.9*	6.2 ± 0.6	6.2 ± 0.7
Peak Pressure (cmH_2_O)	29.80 ± 8.74**	29.6 ± 7.39**	17.90 ± 2.80	17.60 ± 3.34
Plateau Pressure (cmH_2_O)	28.90 ± 8.71**	28.60 ± 7.32**	17.10 ± 3.03	16.70 ± 3.26
PEEP (cmH_2_O)	4.30 ± 0.48	4.30 ± 0.48	4.50 ± 0.52	4.50 ± 0.52
Ventilatory rate (bpm)	18.6 ± 1.4 *	18.7 ± 1.4*	20.7 ± 2.5	21.3 ± 2.2
FIO_2 _(%)	52.50 ± 25.41	48.50 ± 19.58	41.00 ± 2.10	41.00 ± 2.10
pH	7.38 ± 0.04	7.41 ± 0.06	7.39 ± 0.05	7.40 ± 0.04
PaO_2 _(mmHg)	143.60 ± 30.52	133.70 ± 32.92	146.00 ± 81.83	119.80 ± 28.76
PaCO_2 _(mmHg)	34.40 ± 7.32	26.60 ± 5.60	30.29 ± 8.28	31.45 ± 7.18
HCO_3 _(mmol/L)	20.80 ± 6.01	17.89 ± 5.56	19.71 ± 4.58	21.42 ± 5.56
SaO_2 _(%)	98.00 ± 2.58	98.30 ± 2.16	97.60 ± 2.27	97.90 ± 2.18
PaO_2_/FIO_2_	307 ± 101	298 ± 105	358 ± 207	293 ± 74

FIO_2_, inspiratory fraction of oxygen; HCO_3_, bicarbonate; PaCO_2_, partial pressure of carbon dioxide; PaO_2_, arterial oxygen tension; PBW, predicted body weight; PEEP, positive end expiratory pressure; SaO_2_, arterial oxygen saturation; V_T_, tidal volume; *P < 0.01, **P < 0.001 vs. Low V_T _group

**Figure 1:**
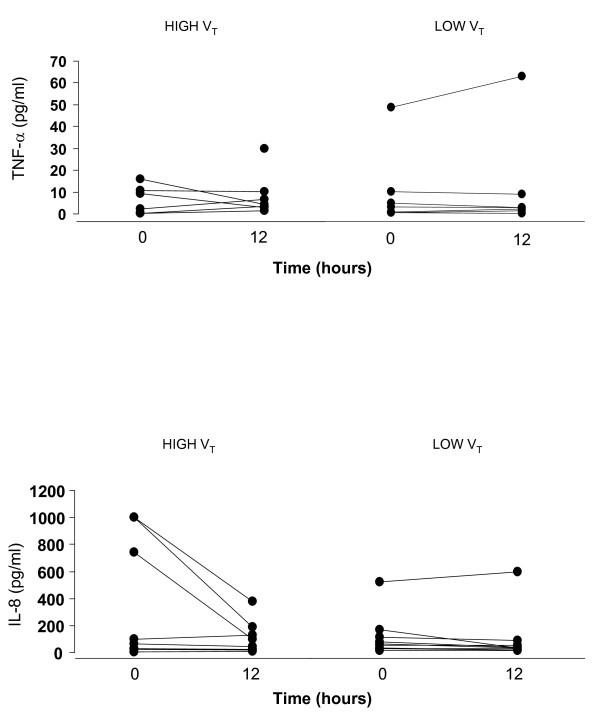
**Time course of plasma TNF-α (top) and IL-8 (bottom) levels in high tidal volume (High V_T_) and low tidal volume (Low V_T_) groups**.

Plasma cytokine levels were similar between the two ventilatory strategies at admission and after 12 h of observation time (Figure 1). Plasma levels of TNF-α remained below the detection limit (0.5 pg/ml) in three patients of the low V_T _group and in two patients of the high V_T _group both at baseline and after 12 h of mechanical ventilation.

At baseline, BAL cytokines concentrations were similar. BAL IL-8 levels in the low V_T _group remained stable (96 (49 to 553) pg/ml vs. 82 (32 to 500) pg/ml, *P *= 0.84) but increased in the high V_T _group (41(10 to 210) pg/ml vs. 328 (50 to 1,000) pg/ml, *P *= 0.01) without differences between groups after 12 hours (*P *= 0.27). After urea standardization, BALF IL-8 values tended to increase in the high V_T _(450 (130 to 20,678) pg/ml vs. 5,000 (2,096 to 14,437) pg/ml, *P *= 0.19) and tended to decrease in the low V_T _(1,809 (735 to 953) pg/ml vs. 1,243 (242 to 2,746) pg/ml, *P *= 0.20) with significant differences 12 hours later (*P *= 0.042). BAL TNF-α decreased in the low V_T _group (12.3 (11.0 to 12.4) pg/ml vs. 6.6 (5.8 to 7.4) pg/ml, *P *= 0.23) but increased in the high V_T _group (1.7 (1.62 to 1.8) pg/ml vs. 22.0 (10.5 to 22.1) pg/ml, *P *= 0.06) with significant differences between groups 12 hours later (*P *= 0.04). Similarly, after urea standardization, BALF TNF-α values tended to decreased in the low V_T _group (177 (90 to 329) pg/ml vs. 87 (34 to 106) pg/ml, *P *= 0.09) but tended to increase in the high V_T _group (49 (21 to 276) vs. 262 (100 to 714), *P *= 0.09) with significant differences between groups 12 hours later (*P *= 0.034).

**Figure 2:**
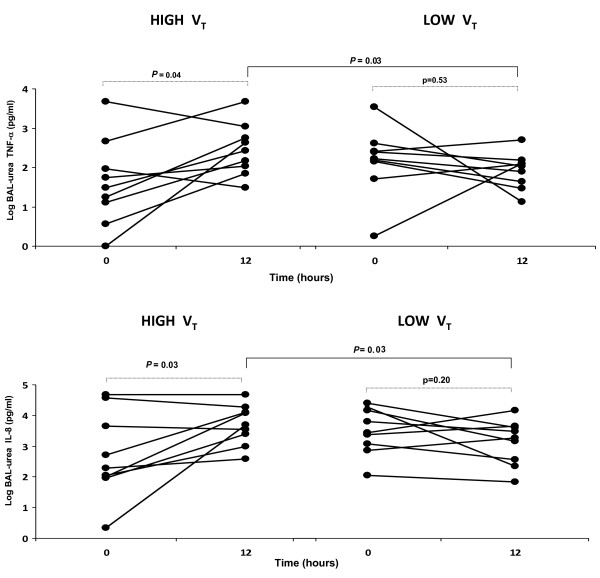
**Time course of logarithmic standardized-urea bronchoalveolar lavage (Log BAL-urea) TNF-α (top) and IL-8 (bottom) levels**. There were no differences between groups at baseline.

After logarithmic transformation, BAL-urea IL-8 values were stable in the low V_T _group (*P *= 0.20) but increased in the high V_T _group (*P *= 0.03) with significant differences after 12 hours (*P *= 0.03). BAL-urea TNF-α values were stable in the low V_T _group (*P *= 0.53) but increased in the high V_T _group (*P *= 0.04) with significant differences after 12 hours (*P *= 0.03) Figure 2.

Due to technical problems, standardization with urea was not possible for one patient in each group.

There was no difference between the two groups with regard to days on mechanical ventilation, intensive care duration of stay, or 28^th ^day mortality rate (Table 1).

## Discussion

The present study has shown that use of lower V_T _and PEEP might attenuate the pulmonary inflammatory response in near normal lungs. The major finding of the study is that both TNF-α and IL-8 concentrations were increased with high V_T _but stable with low V_T _in the BAL fluid in patients ventilated without lung disease after admission to an ICU.

Mechanical ventilation in patients without lung disease is commonly provided by using a V_T _around 10 ml/Kg predicted body weight and a low PEEP [16]. Few studies addressed the effects of mechanical ventilation using a high V_T _strategy on pulmonary inflammatory response in patients without lung disease, mostly during major surgery [11,12,16-18]. In addition, data in non acute lung injury/acute respiratory distress syndrome (ALI/ARDS) ICU patients comes from retrospective analysis [9,19]. Our study distinguishes our study from others as we have shown that protective ventilation in near normal lung patients in an ICU scenario is also beneficial by preventing additional injury.

We observed higher levels of IL-8 and higher TNF-α levels in the BAL fluid of patients ventilated with high V_T _without significant release of lung cytokines into the circulation. Different clinical studies using short periods of mechanical ventilation in patients with normal lungs does not consistently alter plasma levels of inflammatory mediators [11,16,20]. Recently, Wolthuis et al. have shown in patients scheduled to undergo an elective surgical procedure (lasting >5 h) that MV with V_T _of 12 ml/kg and no PEEP increased myeloperoxidase and elastase in the BALF when compared to a V_T _of 6 ml/kg and PEEP but not in plasma inflammatory mediators [13]. In contrast, Michelet et al have shown that protective ventilation reduced the systemic proinflammatory response after esophagectomy [12]. Their results indicate that MV without PEEP and during one lung ventilation is more aggressive to the lungs and can promote a more intense ventilation-induced injury even after a short time. 

Our study differs from most previous studies. We used high V_T _but with PEEP. Therefore, tidal airway closure may be attenuated and gross lung alterations in a short time MV were avoided. The lack of significant systemic or even less intense additional lung inflammation in our study seem to be in accordance to previous research demonstrating initiation of inflammatory responses to injurious ventilatory strategies using high V_T _and ZEEP or low PEEP [1,21-23]. It is known from experimental research that cyclic opening and closing from high V_T _and ZEEP can cause mechanical alterations and histologic damage to peripheral airways and inflammation in lungs [24-27]. Manzano et al. have shown that application of prophylactic PEEP reduces the number of hypoxemia episodes and the incidence of ventilator-associated pneumonia in nonhypoxemic ventilated patients [28].

Although we tried to select patients without acute lung-injury lungs, some degree of inflammation was already present probably due to surgery, trauma, anaesthesia and injurious MV. For instance, we can't exclude that the trans-hiatal manipulation on two patients after esophagectomy did not cause some injury to the lungs. Our results could be explained by a multiple-hit model. Pulmonary inflammation must already be present (first hit) for injurious mechanical ventilation (second hit) to aggravate the inflammatory response. Indeed, both groups had elevated but similar lung cytokine levels at admission with a high tidal volume strategy causing additional increase in most patients, excluding two of them. This hypothesis is supported by experimental studies showing increased inflammatory responses to high V_T _mechanical ventilation after an inflammatory first hit [21,9-31]. There is indeed clinical evidence supporting this multiple-hit hypothesis. High V_T _ventilation was independently associated with development of ARDS in patients who did not have ARDS at the onset of MV in the intensive care unit [9,19]. Furthermore, in patients with acute lung injury or acute respiratory distress syndrome, a protective mechanical ventilation with low V_T _ventilation with PEEP was associated with lower pulmonary and/or systemic inflammatory mediator concentrations, decreased mortality when compared to mechanical ventilation with high V_T_[1,2,4]. 

Our study did not determine the respective influence of a reduced V_T _or lower plateau pressure as both are independently associated with a decrease in ventilator-induced lung injury [32]. Our study design did not produce similar peak and plateau pressures between groups and in the high V_T _group the mean plateau pressure was close to 30 cmH_2_O. Recent data suggest that there is no safe limit for plateau pressure and also for tidal volume [32-34]. Thus, our results can be explained by both low V_T _and plateau pressures.

In our study, the higher ventilatory rate in the low V_T _group narrowed differences in PaCO_2 _between groups and pH was kept absolutely equal. Thus, hypercapnia seems not to explain differences between groups. Hypercapnia may have beneficial physiological and anti-inflammatory effects [35]. However, its role in protective lung ventilation per se, apart from the reduced lung stretch, remains unclear because of lack of clinical data comparing the efficacy of protective lung ventilator strategies in the presence and absence of hypercapnia.

The statistical difference was caused by a majority of patients in the high V_T _group showing increase in BAL cytokines. The non-uniform distribution of these data suggests individual differences in the inflammatory responses. In addition, saline solution instilled and subsequently withdrawn can lead to a variable extent of dilution. The use of urea as a marker of dilution, is based on the knowledge that urea diffuses freely through the alveolar wall. Still after correcting for dilution, cytokines levels showed a non-significant increase in lung cytokines for the high V_T _due to the wide variance that became clearer after logarithmic transformation.

The study was underpowered to correlate outcome variables and release of lung cytokines. The question on the biological role of these mediators in terms of overall inflammatory status or lung injury per se is not easy. There are studies in healthy animals showing that ventilation-induced lung injury might be not caused directly by the mechanical forces, but by the mediators produced in response to these forces [36,37]. Therefore, the interaction between increased cytokines levels caused by injurious ventilation (second hit) and others inflammatory stimuli (first hit) is often difficult to define in a clinical setting.

Additional limitations include the small number of mixed critically ill patients and lack of true blinding. Also, to select near normal lung patients, we planned to include patients without independent predictors for development of acute respiratory distress syndrome such as shock and multiple transfusions [38] or surgeries involving the lungs. Although no patient developed ALI/ARDS during the clinical course, BAL cytokines were elevated and four patients of each group had PaO_2_/FIO_2_ <300 already at baseline. Finally, we did not control ventilation management in the emergency or surgical theatre, but patients met criteria for lungs without acute injury immediately after admission to ICU with study protocol initiation soon after.

## Conclusions

In conclusion, mechanical ventilation with lower V_T _in patients without lung disease resulted in attenuation of pulmonary production of inflammatory mediators. The finding that patients with elevated BAL cytokines levels, immediately before initiation of the protocol, showed higher IL-8 and TNF-α levels during higher V_T _ventilation provides further support to the potential for injurious mechanical ventilation even in patients with previous near normal lungs. Based on our study, we recommend using a protective ventilatory strategy.

## Key messages

• Mechanical ventilation with lower V_T _in patients without lung disease resulted in attenuation of pulmonary production of inflammatory mediators.

• The use of lower tidal volumes may limit pulmonary inflammation in mechanically ventilated patients even without lung injury.

## Abbreviations

ALI: acute lung injury; APACHE: Acute Physiology and Chronic Health Evaluation; ARDS: Acute Respiratory Distress Syndrome; BAL: bronchoalveolar lavage; EDTA: ethylenediaminetetraacetic acid; FIO_2_: O_2 _inspiratory fraction; HCO_3_: bicarbonate; HR: heart rate; IL-8: Interleukin-8; MAP: Mean arterial pressure; PaCO_2_: Partial pressure of carbon dioxide; PaO_2_: partial pressure of arterial oxygen; PBW: Predicted body weight; PEEP: positive end-expiratory pressure; Ppeak: peak inspiratory pressure; Pplateau: plateau pressure after end-inspiratory pause; SaO_2_: arterial oxygen saturation; TE: expiratory time; TI: inspiratory time; TNF-α: tumor necrosis factor-α; VE: minute ventilation; V_T_: tidal volume.

## Competing interests

The authors declare that they have no competing interests.

## Authors' contributions

RPO and GF contributed to the concept, design, and analysis of the study, and drafting of the manuscript. RPO also carried out patient enrolment and coordinated data collection. MPH, MdA and DD assisted with broncoalveolar lavage and data collection.
